# Preferential Subgenome Elimination and Chromosomal Structural Changes Occurring in Newly Formed Tetraploid Wheat—*Aegilops ventricosa* Amphiploid (AABBD^v^D^v^N^v^N^v^)

**DOI:** 10.3389/fgene.2020.00330

**Published:** 2020-05-12

**Authors:** Jie Zhang, Fan Yang, Yun Jiang, Yuanlin Guo, Ying Wang, XinGuo Zhu, Jun Li, Hongshen Wan, Qin Wang, Ziyuan Deng, Pu Xuan, WuYun Yang

**Affiliations:** ^1^Institute of Biotechnology and Nuclear Technology Research, Sichuan Academy of Agricultural Sciences, Chengdu, China; ^2^Key Laboratory of Wheat Biology and Genetic Improvement on Southwestern China (Ministry of Agriculture), Chengdu, China; ^3^Institute of Crop Research, Sichuan Academy of Agricultural Sciences, Chengdu, China; ^4^Institute of Agro-products Processing Science and Technology, Sichuan Academy of Agricultural Sciences, Chengdu, China

**Keywords:** tetraploid wheat, *Aegilops ventricosa*, amphiploid, chromosomal variation, mc-FISH

## Abstract

Artificial allopolyploids derived from the genera *Triticum* and *Aegilops* have been used as genetic resources for wheat improvement and are a classic example of evolution via allopolyploidization. In this study, we investigated chromosomes and subgenome transmission behavior in the newly formed allopolyploid of wheat group via multicolor Fluorescence *in situ* hybridization (mc-FISH), using pSc119.2, pTa535, and (GAA)_7_ as probe combinations, to enabled us to precisely identify individual chromosomes in 381 S_3_ and S_4_ generations plants derived from reciprocal crosses between *Ae. ventricosa* (D^v^D^v^N^v^N^v^) and *T. turgidum* (AABB). A higher rate of aneuploidy, constituting 66.04–86.41% individuals, was observed in these two early generations. Of the four constituent subgenomes, D^v^ showed the highest frequency of elimination, followed by N^v^ and B, while A was the most stable. In addition, structural chromosomal changes occurred ubiquitously in the selfed progenies of allopolyploids. Among the constituent subgenomes, B showed the highest number of aberrations. In terms of chromosomal dynamics, there was no significant association between the chromosomal behavior model and the cytoplasm, with the exception of chromosomal loss in the D^v^ subgenome. The chromosome loss frequency in the D^v^ subgenome was significantly higher in the *T. turgidum* × *Ae. ventricosa* cross than in the *Ae. ventricosa* × *T. turgidum* cross. This result indicates that, although the D subgenome showed great instability, allopolyploids containing D subgenome could probably be maintained after a certain hybridization in which the D subgenome donor was used as the maternal parent at its onset stage. Our findings provide valuable information pertaining to the behavior patterns of subgenomes during allopolyploidization. Moreover, the allopolyploids developed here could be used as potential resources for the genetic improvement of wheat.

## Introduction

Common wheat, or bread wheat, (*Triticum aestivum* L, AABBDD) is one of the most important food crops worldwide, with extensive reports on its speciation. To date, it has been widely accepted that two sequential allopolyploidization events occurred during evolution. The first allopolyploidization occurred about 0.3–0.5 million years ago, involving interspecific hybridization between *Triticum urartu* (AA) and an unknown *Aegilops* species, possibly related to *Aegilops speltoides* (SS), which led to the formation of emmer wheat (AABB) (Dvorak and Zhang, 1990; Huang et al., 2002; EI El Baidouri et al., 2017). Subsequently, allopolyploidization between tetraploid wheat (AABB) and *Aegilops tauschii* (DD) occurred about 10,000 years ago, which gave rise to the speciation of modern bread wheat (AABBDD) (Kilian et al., 2007; Marcussen et al., 2014). As a classical sample for the survey of evolution via allopolyploidization, several studies exist on chromosomal behavioral patterns in nascently synthesized allopolyploids of the *Triticum* tribe, including the crossing of tetraploid or hexaploid wheat with *Aegilops* and *Secale* species. For example, Zhao et al. (2011) observed that an individual plant harbored 50 chromosomes in the S_2_ generation that were derived from a newly formed allohexaploid wheat line (2n = 42, AABBDD). Various frequencies of aneuploidy were detected in the S_1_ and S_2_ generations derived from the crosses between several genotypes of *Triticum durum* (AABB) and *Ae. tauschii* (DD) (Mestiri et al., 2010), and persistent aneuploidy was found to be associated with nascent allohexaploid wheat (AABBDD) in the S_1_ to >S_20_ generations (Zhang et al., 2013a). In addition to variation in chromosome numbers (chromosome loss/gain), extensive chromosome rearrangements, scale genomic changes of repetitive DNA, and copy-number variations in gene homologs were detected in four newly synthesized allotetraploid wheat lines (genome compositions were S^sh^S^sh^A^m^A^m^, S^l^S^l^AA, S^b^S^b^DD, and AADD, respectively) (Zhang et al., 2013b). Telosome mutations were also observed in a newly formed hexaploid wheat derived from a cross between *Triticum turgidum ssp. dicoccum* MY3478 (AABB) and *Ae. tauschii* SY41(DD) (Li H. et al., 2016). Among the constituent subgenomes, chromosomal instability exhibiting obvious subgenome-bias was observed. Most of the previous studies demonstrated a preferential elimination of the D subgenome compared to the A and B subgenome, and even the R, S, U, and Ns genome (Tiwari et al., 2010; Xie et al., 2012; Hao et al., 2013; Li et al., 2015; Guo et al., 2018). Mestiri et al. (2010) proposed that genome stability was dependent on the genotypes of both A, B, and D genome donors. Nonetheless, chromosomal alterations in the D subgenome, which are not directly contributed by wheat or *Ae. tauschii*, have not been investigated in detail, and it remains uncertain whether the chromosome behavior pattern exhibits cytoplasm-dependence.

*Aegilops ventricosa Tausch* (2n = 28, genome D^v^D^v^N^v^N^v^) has potential as a genetic source of germplasm due to its ability to tolerate biotic stresses and may be useful for wheat improvement. In recent decades, two sets of *Ae. ventricosa* introgression lines, H-93 and VPM1 derived from the interspecific hybridization of *T. aethiopicum* with *Ae. ventricosa* (accession AP-1) and *T. persicum* with *Ae. ventricosa* (accession #10), respectively (Maìa, 1967; Dosba et al., 1978), have been studied using cytogenetic methods and widely used in wheat improvement. Until this, H-93, VPM1, and their derivative lines have demonstrated resistances against several wheat pathogens, such as powdery mildew (Delibes et al., 1987), eyespot (*Pch1*) (Doussinault et al., 1983), rust (*Lr37*, *Yr17*, and *Sr38*) (Bariana and McIntosh, 1993, 1994; Bonhomme et al., 1995; Tanguy et al., 2005), cereal cyst nematode (*Cre2*, *Cre5*, and *Cre6*) (Delibes et al., 1993; Jahier et al., 2001; Ogbonnaya et al., 2001; Tanguy et al., 2005), and Hessian fly (*H27*) (Delibes et al., 1997). In order to effectively exploit the genetic value originating from *Ae. ventricosa*, novel *Ae. ventricosa* accessions are needed to be employed in the development of wheat-*Ae. ventricosa* introgression lines.

In this study, we used multicolor FISH (mc-FISH) to investigate 381 experimental plants, covering S_3_ and S_4_ generations derived from reciprocal crosses between *Ae. ventricosa* (D^v^D^v^N^v^N^v^) and *T. turgidum* (AABB). Our aim was to characterize the chromosome alterations in nascent wheat allopolyploids for use as novel genetic resources of *Ae. ventricosa*. As a result, chromosome alterations were found to be accompanied by the formation of the new allopolyploids. Subgenome and chromosome biases were also observed. No association was observed between chromosomal dynamics and cytoplasm, with the exception of the chromosomal loss in the D^v^ subgenome.

## Materials and Methods

### Plant Materials

**FIGURE 1:**
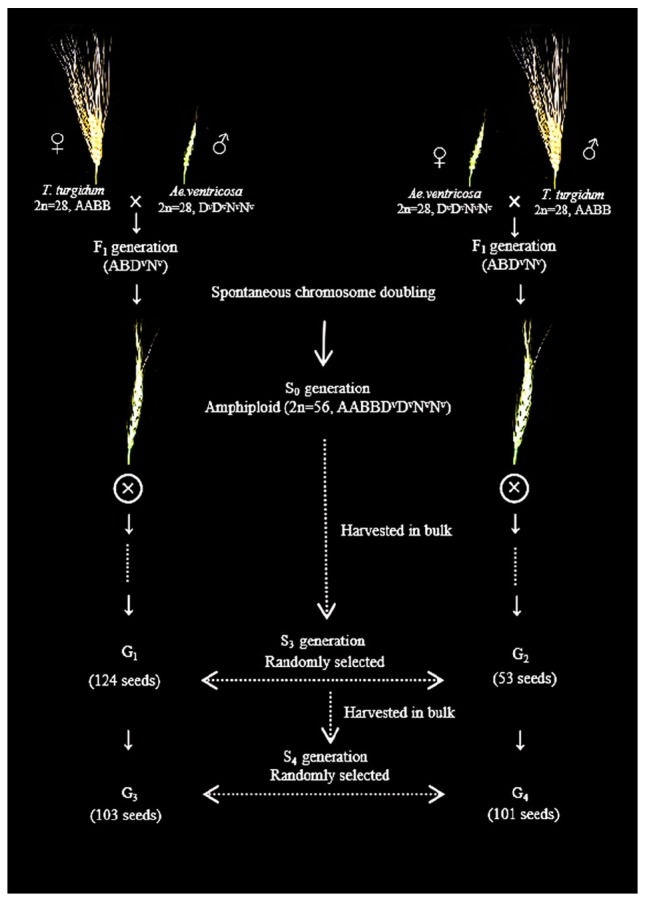
Schematic of strategy used to obtain the experimental seeds (G_1_, G_2_, G_3_, and G_4_) using hybridization between *T. turgidum* and *Ae. ventricosa.*

*Aegilops ventricosa* cv. RM271 (D subgenome of *Ae. ventricosa*, or D^v^) was supplied by Prof. Lihui Li (Institute of Crop Sciences, Chinese Academy of Agricultural Sciences). *Aegilops tauschii* SQ 665 (D subgenome of *Ae. tauschii*, or D^t^) was provided by International Maize and Wheat Improvement Center (CIMMYT). *T. turgidum* var. *durum* cv. Langdon and common wheat Chinese Spring (CS) (D subgenome of bread wheat, or D^b^) were conserved by our library. These four experimental lines were used to develop a standard FISH karyotype of A, B, D^v^, D^t^, D^b^, and N^v^ subgenomes. Spontaneous amphidiploids of *T. turgidum*–*Ae. ventricosa* were obtained from fertile F_1_ generations of *Ae. ventricosa* cv. RM271 (as female or male) crossed with *T. turgidum* cv. Langdon (*T. turgidum* × *Ae. ventricosa*; *Ae. ventricosa* × *T. turgidum*) via chromosome autoduplication. The seeds of S_3_ [derived from *T. turgidum* × *Ae. ventricosa* (group 1, or G_1_) and *Ae. ventricosa* × *T. turgidum* (group 2, or G_2_)] and S_4_ [derived from *T. turgidum* × *Ae. ventricosa* (group 3, or G_3_) and *Ae. ventricosa* × *T. turgidum* (group 4, or G_4_)] generations were collected. Then, 124, 53, 103, and 101 seeds (381 seeds in total) were randomly selected from G_1_, G_2_, G_3_, and G_4_, respectively, for analysis of the FISH karyotype (Figure 1).

### mc-FISH Procedures

The root tips used for karyotype analyses were collected from germinating seeds and treated with nitrous oxide. The protocol was conducted as described by Zhang J. et al. (2018) The chromosomal preparation of mitotic metaphases was performed as reported by Kato et al. (2004). To identify every pair of chromosomes originating from *T. turgidum* and *Ae. ventricosa*, oligo-nucleotide probes Oligo-pSc119.2, Oligo-pTa535, and Oligo-(GAA)_7_ were labeled on the 5′-end with 6-carboxyfluorescein (6-FAM), 6-carboxytetramethylrhodamine (Tamra), and Cy5, respectively, and used as described by Tang et al. (2014) and Li G. et al. (2016) (Invitrogen, Shanghai, China). FISH protocols were adapted from the methods described by Han et al. (2006). The slides were stored in a moist box at 37°C for 4 h and washed in 2 × SSC at room temperature. The slides were mounted with VECTASHIELD mounting medium with 4′,6-diamidino-2-phenylindole (DAPI) (Vector Laboratories, CA, United States). Images were captured using a Leica DM2500 fluorescence microscope (Leica, Wetzlar, Germany) equipped with a cooled charge-coupled device camera (Leica, Wetzlar, Germany) operated with LAS Live software (version 4.6) (Leica, Wetzlar, Germany). Signal pattern images for the same metaphase cell were taken over two rounds. The first round was used to capture the signals of probes labeled with 6-FAM (Oligo-pSc119.2) and Tamra (Oligo-pTa535), which were displayed green and red and the chromosomal background stain, DAPI, which was displayed as blue; the three resulting images (Oligo-pSc119.2, Oligo-pTa535 and DAPI) were merged. In the second round, the signals of Oligo-(GAA)_7_ labeled with Cy5 and the chromosomal background stain, DAPI, which displayed yellow and red, respectively, were obtained; the two resulting images (Oligo-(GAA)_7_ and Cy5) were merged.

### Statistics Analysis

Statistical analyses were performed using SPSS version 19.0 (IBM, United States) and GraphPad Prism version 8.01 (GraphPad Software, United States). Chi-square test or Student’s *t*-test were used to estimate the statistical significance for each comparison with a *p*-value 0.05 as the threshold.

## Results

### Amphiploid Plant Morphology (S_1_ Generation)

**FIGURE 2:**
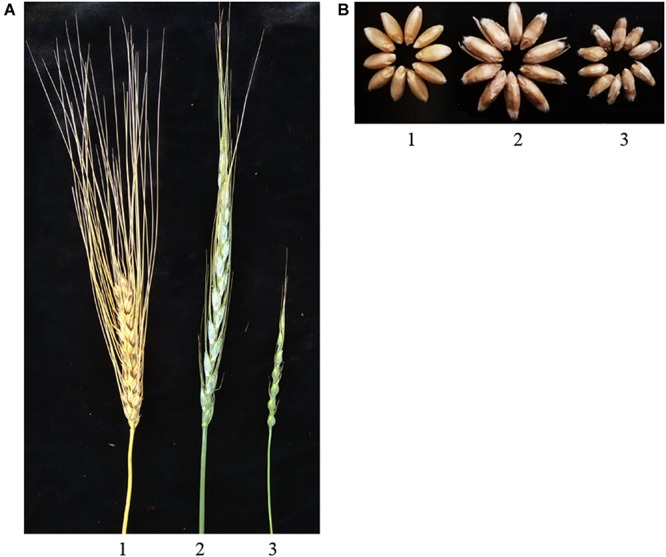
Spike **(A)** and seeds **(B)** morphology. 1–3 are *T. turgidum* var. *turgidum* cv. Langdon, amphiploid, and *Ae. ventricosa* cv. RM271, respectively.

Phenotypically, amphiploids exhibited a spike-shape similar to that of their parents *Ae. ventricosa.* The spike-lengths of amphiploids were clearly longer than those either of the parents, *T. turgidum* and *Ae. ventricosa.* The short awns of the spikes in the progenies were likely inherited from *Ae. ventricosa* (Figure 2A). In addition to the spikes, the length and width of the amphiploids seeds were higher than those of either of the parents. Moreover, the amphiploids had tough glumes similar to those of the parent *Ae. ventricosa*, which made the spikes difficult to thresh (Figure 2B).

### FISH Karyotypes of A, B, D^v^, N^v^ Subgenomes Were Established Using the Probe Combination of Oligo-pSc119.2, Oligo-pTa535, and Oligo-(GAA)_7_

**FIGURE 3:**
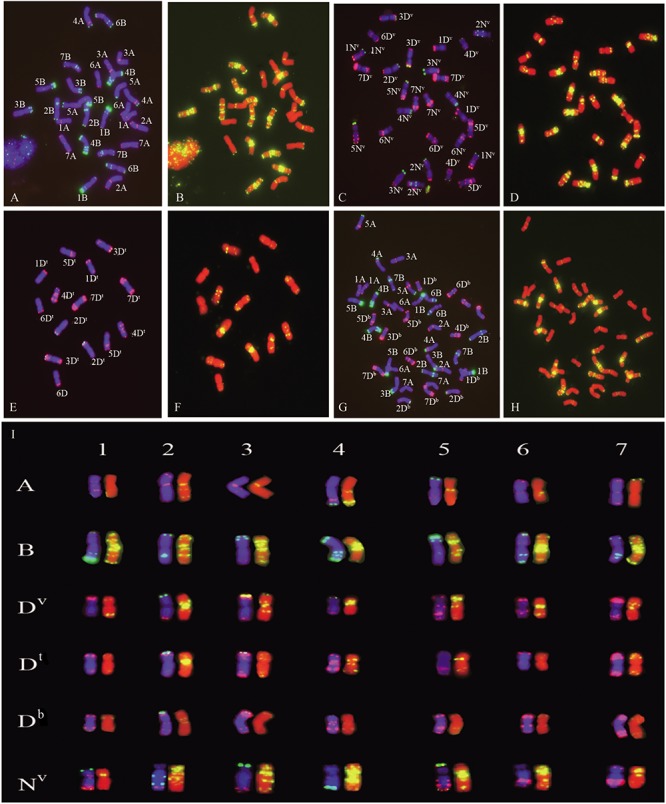
FISH analysis of *T. turgidum* cv. Langdon **(A,B)**, *Ae. ventricosa* cv. RM271 **(C,D)**, *Ae.tauschii* SQ 665 **(E,F)**, common wheat Chinese Spring (CS) **(G,H)**, and the karyograms of the A, B, D^v^, D^t^, D^b^, and N^v^ subgenomes **(I)**. **(A,C,E,G)** chromosomes stained by DAPI (blue), oligo-nucleotides Oligo-pSc119.2 (green), and Oligo-pTa535 (red). **(B,D,F,H)** Chromosomes staining with DAPI (red) and Oligo-(GAA)_7_ (yellow). A, B in panel **(I)** are the subgenomes of *T. turgidum* cv. Langdon; D^v^ and N^v^ are the subgenomes from *Ae. ventricosa* cv. RM271; D^t^ is the genome originated from *Aegilops tauschii* cv. SQ 665; D^b^ is the subgenome from bread wheat Chinese Spring (CS).

The chromosomes of the parents *T. turgidum* and *Ae. ventricosa* were analyzed by mc-FISH using a combination of probes Oligo-pSc119.2, Oligo-pTa535, and Oligo-(GAA)_7_. As shown in Figures 3A–D, I, Oligo-pSc119.2 was mainly localized to the B and N^v^ genomes, while high levels of Oligo-pTa535 signal were observed on A and D^v^ genomes. Oligo-(GAA)_7_ exhibited strong signals in the centromeric regions of all the B chromosomes and large parts of the D^v^ and N^v^ chromosomes. The integrated use of probes allowed for the identification of the individual chromosomes of the A, B, D^v^, and N^v^ genomes. *Ae. ventricosa* is a tetraploid originating from of *Ae. tauschii* and *Ae. uniaristata* (NN). The D^v^ genome of *Ae. ventricosa* (D^v^D^v^N^v^N^v^) is similar to that of *Ae. tauschii* (DD) (McNeil et al., 1994), which allowed us to precisely identify each pair of D^v^ and N^v^ chromosomes according to the FISH karyotype of *Ae. tauschii* and the descriptions reported by Badaeva et al. (2011). Comparison of the FISH karyotypes of the D^v^ genome with that of D^t^ genome revealed significant differences in terms of the microsatellite sequence distribution patterns, based on the signal distribution of Oligo-(GAA)_7_. For example, 1D^v^ harbored strong and weak signal bands at the end of long and short arms, respectively, while 1D^t^ showed weak signal bands at both ends of the long and short arms; 3D^v^ carried signals at the centromeric region and the end of short arm, while 3D^t^ carried signals at the centromeric region and the end of the long arm; 4D^v^ showed a stronger signal than 4D^t^; 6D^v^ had an obvious signal at the centromeric region and sub-terminal region of the long arm. Conversely, 6D^t^ showed the extremely low levels of signal along the chromosome; 7D^v^ showed signal at the centromeric region, whereas 7D^t^ showed signal at the terminal region of the chromosome. Compared to the D^v^ and D^t^ genomes, extremely low levels of signal of Oligo-(GAA)_7_ was detected along the all chromosomes from the D^b^ genome of bread wheat, Chinese Spring (CS) (Figures 3C–I). These results indicated that the signal patterns of Oligo-(GAA)_7_ of D^v^ and D^t^ were similar to those of D^b^ (Figure 3I).

### High Levels of Whole-Chromosome Aneuploidy in Early Generations

**FIGURE 4:**
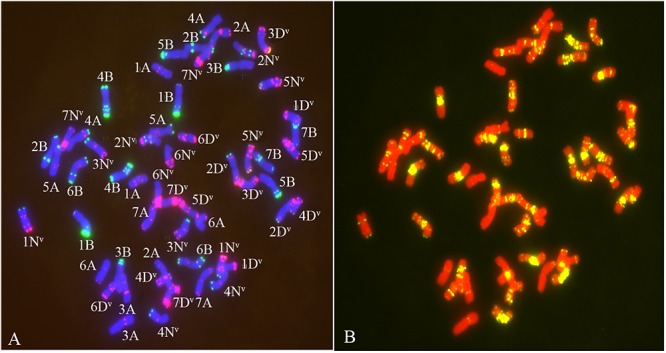
The FISH pattern from euploids. **(A)** Chromosomes staining with DAPI (blue) oligo-nucleotides Oligo-pSc119.2 (green), Oligo-pTa535 (red). **(B)** chromosomes staining by DAPI (red) and Oligo-(GAA)_7_ (yellow).

**FIGURE 5:**
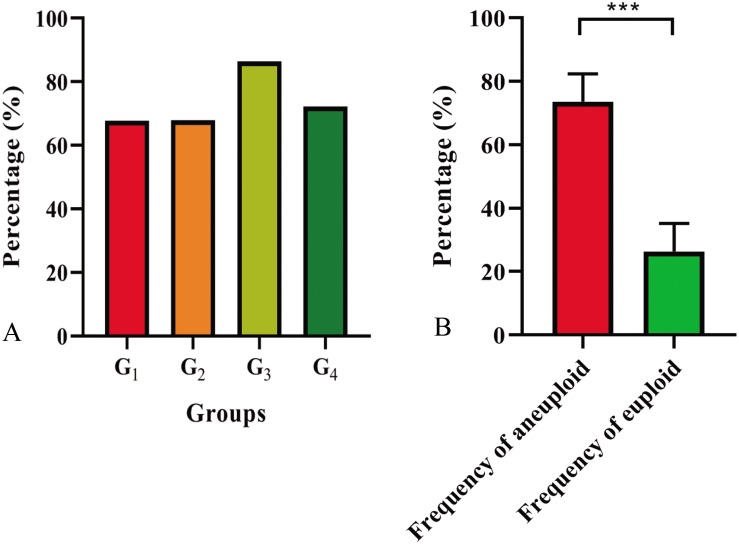
Aneuploid distribution **(A)** in four groups and frequencies of aneuploids and euploids **(B)**. *P*-values labeled with asterisks denote significant difference at ***P < 0.001.

**FIGURE 6:**
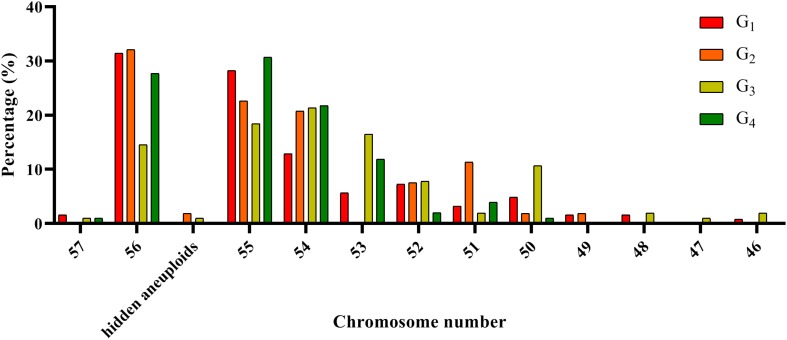
Distribution of the number of chromosomes in the four experimental groups.

The chromosome composition of the 381 individuals from the four groups were analyzed via mc-FISH. All of the mitotic cells investigated showed the same FISH karyotype, indicating that no somatic alteration existed among the cells from the same individuals. A small proportion of euploids were identified (Figure 4), and high levels of whole-chromosome aneuploidy were observed in all groups, with frequencies varying from 67.74 to 86.41% (Figure 5A). The frequency of aneuploids was significantly higher than that in euploids (*t*-test, *p* = 0.000) (Figure 5B). All four groups showed variable chromosome numbers and the chromosome numbers of the experimental plants ranged from 46 to 57 (Figure 6 and Supplementary Figure S1). Of the 381 plants, the frequency of plants exhibiting chromosomal loss was prominent compared to those exhibiting chromosomal gain, with rates of 72.23 and 4.46%, respectively.

**FIGURE 7:**
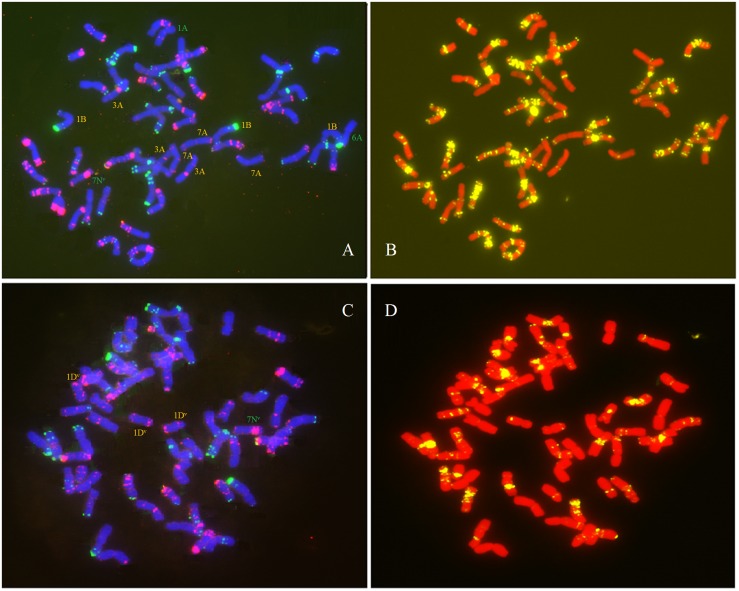
FISH analysis of two types of “hidden aneuploidy” using Oligo-pSc119.2 (green), Oligo-pTa535 (red) and Oligo-(GAA)_7_ (yellow) as probes. **(A,B)** 17Y-44-95; **(C,D)** Y1701-1-2. Green denotes chromosome loss and yellow denotes chromosome gain.

We observed a special type of aneuploidy in G_2_, and G_3_. This type of aneuploidy contained 56 chromosomes, as in euploids, but exhibited chromosome loss and gain, which was denoted as hidden aneuploidy by Zhang et al. (2013a). Of the 381 plants, two plants (0.52%) were classified as having “hidden aneuploidy,” 17Y-44-95 (in G_2_) and Y1701-1-2 (in G_3_) (FISH signal patterns are shown in Figures 7 A–D). Y1701-1-2 involved 7N loss/1D gain, while 17Y-44-95 showed a complex “hidden aneuploidy” pattern involving 1A, 6A, 7N loss/7A, 3A, 1B gain. Notably, the two “hidden aneuploidy” lines involved chromosomes (loss/gain) originating from different homologous groups.

**FIGURE 8:**
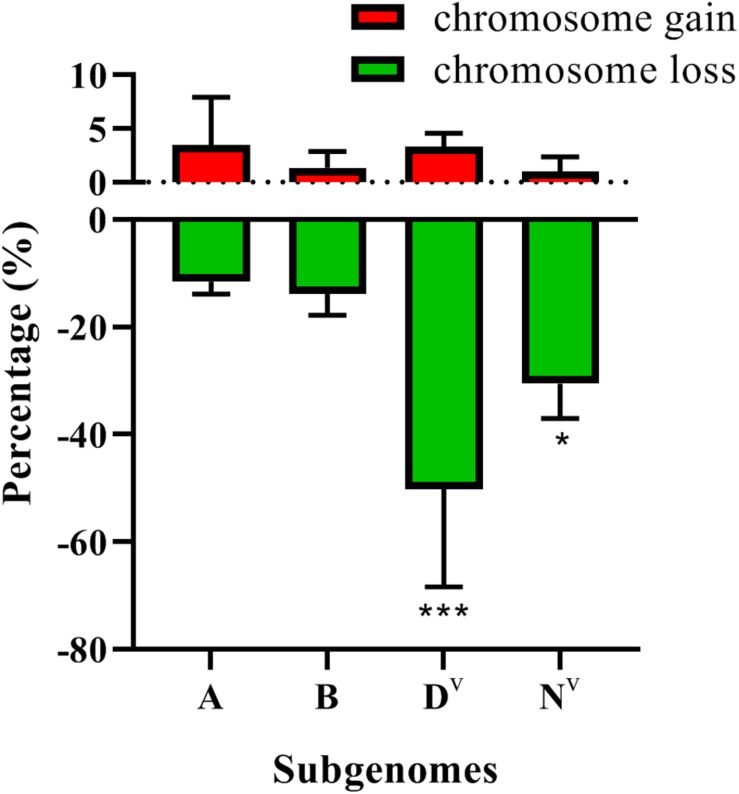
The frequencies of subgenome loss and gain. *P*-values labeled with asterisks denote significant differences at *P < 0.05 and ***P < 0.001.

These results indicate a large incidence of aneuploidy in the early generations of the neoallopolyploids resulting from the *T. turgidum* × *Ae. ventricosa* cross. Subsequently, we explored whether chromosome loss/gain events were associated with genome biases. D^v^ subgenome was found to show the highest frequency of chromosomal loss (50.27%) (Chi-square test, *p* = 0.00). The N^v^ subgenome (30.59%) showed a significantly higher frequency of chromosomal loss than the B (13.81%) or A (11.61%) subgenomes (Chi-square test, *p* = 0.017, N^v^ vs. B; Chi-square test, *p* = 0.02, N^v^ vs. A), while no significant differences were detected between the A and B subgenomes (Chi-square test *p* = 0.415) (Figure 8). In terms of chromosome gain, A and D^v^ showed the highest frequency, 3.48 and 3.29%, respectively, while N^v^ showed the lowest frequency of chromosomal gain (0.99%). However, no significant differences related to chromosomal gains were found among the four subgenomes (Chi-square test, *p* > 0.05) (Figure 8).

### Incidence of Chromosome Structural Variations and Different Propensities Among Constituent Genomes

Compared to variations in chromosome numbers, variations in chromosome structures occurred in a smaller proportion. Among the 381 plants, changes in chromosome structures were observed in 39 individuals, with a rate of 10.24%, including 47 events of chromosomal breakage. Among these 39 plants, two types of chromosomal structural variation were detected: chromosomal breakage (including 1AS. 1AL-, 3AL, -4AS.4AL, 6AL, 1BL, 2BS. 2BL-, 4BL, 5BS, -6BS.6BL, 7BS. 7BL-, 2D^v^L, 3D^v^L, 6D^v^S.6D^v^ L-, 1N^v^L, 3N^v^S.3N^v^ L-, and 6N^v^S) and translocation (including 3BL.3BL, 5N^v^L.5N^v^L, 7A.7D, 5AL.5AL, and 7N^v^L.3BL).

**FIGURE 9:**
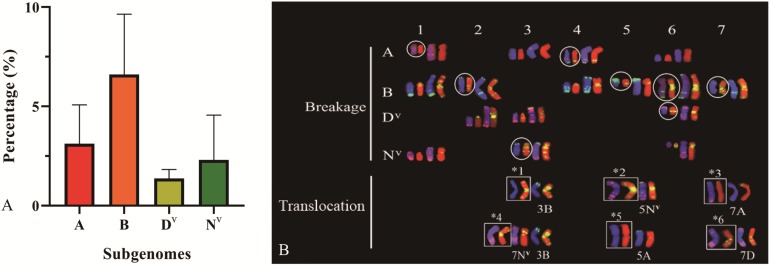
The distribution of chromosomal structural variations of four subgenome **(A)**, and the FISH karyotype of chromosomal structural changes **(B)** by use of chromosomal structural variations. ^∗^1, 3BL.3BL; ^∗^2, 5N^v^L.5N^v^L; ^∗^3, 7AS.7D^v^L; ^∗^4, 7N^v^L.3BL; ^∗^5, 5AL.5AL; ^∗^6, 7D^v^S.7AL. Circles denote chromosomes with breakages occurring in non-centromeric region.

All four subgenomes (A, B, D^v^, and N^v^) underwent chromosome structural variation, and 11 (2.89%, A subgenome), 23 (6.04%, B subgenome), five (1.31%, D^v^ subgenome), and eight (2.10%, N^v^ subgenome) plants carrying chromosomal structural change, respectively, were observed in 381 plants (Figure 9A). Similar to the variation in chromosome numbers, chromosomal structural variations were accompanied by genome bias. Among the four constituent subgenomes, B subgenome showed significantly higher frequency of structural variations (Chi-square test, *p* = 0.048, B vs. A; *p* = 0.005, B vs. D^v^; *p* = 0.016, B vs. N^v^). A large proportion of chromosomes were observed to exhibit structural variations from all four subgenomes, while no visible structural variations were detected on chromosomes 2A, 1D^v^, 4D^v^, 5D^v^, 2N^v^, and 4N^v^ (Figure 9B and Supplementary Figure S2).

### Significant Differences in Chromosomal Behavior Model Between Progenies From Reciprocal Crosses Observed Only in the Chromosome Loss of the D^v^ Subgenome

**FIGURE 10:**
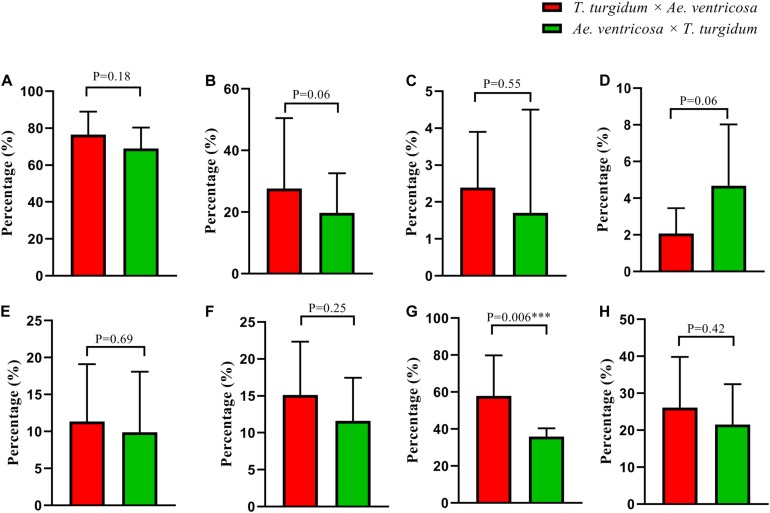
Chromosomal behavior biases in reciprocal crosses. **(A)** Frequencies of aneuploids; **(B)** Frequencies of chromosome loss; **(C)** Frequencies of chromosome gain; **(D)** Frequencies of chromosomal structural variation; **(E–H)** Frequencies of chromosome loss in A, B, D^v^, and N^v^ subgenome.

In terms of the aneuploidy frequency and subgenome bias of chromosomal numerical/structural variation, no significant differences were found between the tetraploid wheat cytoplasm and *Ae. ventricosa* cytoplasm (Figures 10A–F,H). Interestingly, a significant difference was detected for chromosome loss in D^v^ subgenome between the reciprocal crosses (*t*-test, *p* = 0.006 for *T. turgidum* × *Ae. ventricosa* vs. *Ae. ventricosa* × *T. turgidum*) (Figure 10G). However, because the frequencies were low, we were unable to conduct a statistical analysis for chromosome gain and chromosomal structural variations of the four constituent subgenomes.

## Discussion

Repetitive sequences have significantly contributed to our understanding of genome organization and recombination during the evolution of plants (Friesen et al., 2001). Numerous FISH probes have been developed based on repetitive sequences (Cuadrado and Jouve, 2010; Tang et al., 2014, 2016, 2018; Fu et al., 2015; Lang et al., 2018; Liu et al., 2018), which are widely used for the identification of chromosomes of the *Triticum* genus (Badaeva et al., 2011; Zhang et al., 2013b; Delgado et al., 2016; Zhao et al., 2018; Ren et al., 2019). In this study, we established the FISH karyotypes of the A, B, D^v^, and N^v^ subgenomes using Oligo-pSc119.2, Oligo-pTa535 and Oligo-(GAA)_7_. As expected, these probes distinguished between the A, B, D^v^, and N^v^ subgenomes. Moreover, the signal patterns on the chromosomes of the A and B subgenomes were consistent with the results reported by Tang et al. (2014). Since *Ae. ventricosa* originated from the hybridization of *Ae. tauschii* (DD) and *Ae. uniaristata* (NN) (McNeil et al., 1994), each individual chromosome from D^v^ subgenome could be identified according to the FISH signal patterns of D genome of *Ae. tauschii*.

In the present study, we compared the FISH karyotypes of the three types of D genomes (D^t^ genome of *Ae. tauschii*, D^v^ of *Ae. ventricosa*, and D^b^ of *T. aestivum*), and we found that the three D genomes showed slight differences within themselves. These results indicated that D genome present in *Ae. ventricosa* (D^v^) and *T. aestivum* (D^b^) underwent small modifications compared to the ancestral D genomes of *Ae. tauschii*, which is in agreement with the results reported by Mirzaghaderi and Mason (2017). Notably, we found that the three D genomes showed similar FISH signal patterns for the Oligo-pSc119.2 and Oligo-pTa535 probes compared to the Oligo-(GAA)_7_ probe, which indicated that the tandem repeat sequences of pSc119.2 (KF719093) and pTa535 (KC290894.1) were likely to be more conserved than simple repeat sequences (GAA)_*n*_ over evolutionary time. Interestingly, the 1D^v^–7D^v^, 2D^t^–5D^t^, and 7D^t^ chromosomes showed observable levels of Oligo-(GAA)_7_ signal patterns. Specifically, 2D^v^ and 2D^t^, 4D^v^ and 4D^t^, and 5D^v^ and 5D^t^ shared similar Oligo-(GAA)_7_ signal patterns. However, the chromosomes of D^b^ subgenome showed fewer and lower levels of Oligo-(GAA)_7_ signals compared to the chromosomes of the D^v^ and D^t^ subgenomes. The FISH signal patterns of Oligo-(GAA)_7_ on the chromosomes of the D^v^ and D^t^ subgenomes were more similar than those of the chromosomes of the D^b^ and D^t^ subgenomes. These results showed a burst of simple repeat sequences occurred on both D^v^ and D^t^ subgenomes rather than D^b^ subgenome, implying that the divergence between the D^t^ of *Ae. tauschii* and D^v^ of *Ae. ventricosa* most likely occurred later than between D^t^ of *Ae. tauschii* and D^b^ of bread wheat, indicating that intraspecific hybridization of *Ae. tauschii* and *Ae. uniaristata* (NN) may have occurred later than the intergeneric hybridization of *Ae. tauschii* and emmer wheat.

Studies on the behavior of subgenomes and single chromosomes concerning the last post-allopolyploidization timescale are valuable to improve our understanding of the formation and evolution of allopolyploids. Recently, many studies have demonstrated that allopolyploidization in the wheat group triggered extensive genomic shocks, which led to karyotype changes, including changes in chromosome structure and numbers (Hao et al., 2013; Zhang et al., 2013a; Li G. et al., 2016; Guo et al., 2018). Large and frequent whole chromosome loss/gain events and chromosome/subgenome biases resulting from aneuploidy in the neoallopolyploids of the wheat group have been described in detail in the literature (Tiwari et al., 2010; Zhou et al., 2012; Hao et al., 2013; Zhang et al., 2013b; Li et al., 2015). However, the majority of the literature is only concerned with neoallopolyploids harboring the D subgenome originating from *Ae. tauschii* or *T. aestivum.* As a result, neoallopolyploids carrying other types of D subgenome originating from *Aegilops* genus have not been fully investigated; whether the maternal parent effect the chromosomal behavior remains unknown.

We addressed these issues using two sets of newly formed *T. turgidum*-*Ae. ventricosa* allopolyploidy (AABBD^v^D^v^N^v^N^v^) lines derived from reciprocal crosses between *Ae. ventricosa* and *T. turgidum.* Many aneuploids were observed in the S_3_ (G_1_ and G_2_) and S_4_ (G_3_ and G_4_) generations, with frequencies of 67.74, 67.92, 86.41, and 72.28%, respectively, which correlate with the results of several recent studies (Zhang et al., 2013b; Guo et al., 2018). It is widely believed that aneuploidy generally occurs in newly formed allopolyploid wheat lines. However, natural wheat varieties show a much lower frequency of aneuploid individuals (1–3%) (Riley and Kimber, 1961). Recently, several studies have attempted to address the underlying mechanisms of stability that give rise to the formation of allopolyploid in wheat. For example, Zhang et al. (2013b) found that persistent aneuploidy is generally associated with nascent allohexaploid wheat over multiple generations (S_1_–S_20_) and further proposed that karyotypic stabilization could not be achieved even by consecutive screening for euploidy. Mestiri et al. (2010) demonstrated that the stability of synthetic allohexaploids in wheat was dependent on the variability of genotypes of *T. turgidum* and *Ae. tauschii.* Our findings showed that the cytoplasm of the maternal parent did not affect the frequency of euploidy in the wheat group, which suggests that many aneuploids would occur in early generations no matter which plant was used as the maternal parent. Thus, the key constituent affecting the stabilization of allopolyploids in natural wheat also warrants further investigation. Given the much higher rate of the plants harboring chromosome loss (72.23%) compared to chromosome gain (4.46%), it was obvious that chromosome loss would occur more easily than chromosome gain, which most likely depends on the capacity of cell nucleus *per se*.

Chromosomal aneuploidy is known to exhibit preferential chromosome elimination. Our results demonstrated that the D^v^ subgenome showed a higher frequency of loss (50.27%) and gain (3.29%) than the other three constituent subgenomes, suggesting that D^v^ is the most unstable subgenome of our newly formed allopolyploids. The result is in agreement with a significant number of previous studies, such as wheat-*Secale* (RR) allopolyploids (A, B, D, and R) (Dou et al., 2006; Zhou et al., 2012; Hao et al., 2013; Li et al., 2015), wheat-*Ae.kotschyi* (U^k^U^k^S^k^S^k^) allopolyploids (A, B, D, U^k^, and S^k^) (Tiwari et al., 2010), newly formed wheat allotetraploids (AADD) (Guo et al., 2018), and trigeneric hybrids (A, B, D, R, and Ns) of wheat–*Secale* (RR)–*Psathyrostachys huashanica* (NsNs) (Xie et al., 2012), but contrary to the “pivotal-differential genome evolution hypothesis”(Mirzaghaderi and Mason, 2017), which proposed that D, A, and U were pivotal genomes, and underwent little modifications. Thus, they were considered to be more stable than under-dominant subgenomes (the other subgenomes harbored in the wheat group with the exception of A, D, and U). Possibly, this mechanism may have given rise to the formation of allohexaploid wheat over evolutionary time (Zhang et al., 2013a). There is significant evidence that has supported pivotal-differential genome patterns as a common phenomenon during the evolutionary process of wheat group via allopolyploidization. For example, allotetraploids *Ae. crassa* (DM), *Ae. cylindrica* (DC), and *Ae. triuncialis* (UC) showed lower amounts of sequence loss in their pivotal subgenomes (D and U) than in their differential subgenomes (M and C) (Senerchia et al., 2014); in terms of allohexaploid bread wheat, the higher sequence order conservation was detected in the A genome relative to B and in the D genome compared to A and B (Pont et al., 2013); in allohexaploid *Ae. neglecta* (2n = 6 × = 42, U^n^U^n^M^n^M^n^N^n^N^n^), U (pivotal subgenome) has remained mostly intact relative to M and N (differential subgenome) (Badaeva et al., 2004). Integrating our findings with the previous results, we propose that in early generations of newly resynthesized allopolyploids of wheat group, chromosomal behavior pattern probably may not strictly follow this rule. The D subgenome is likely to be more easily “shocked” than other subgenomes (including pivotal subgenomes, A, B, and U, even the differential subgenomes S, R, N, and Ns) once they are merged with other genomes, which leads to its preferential elimination. The preferential elimination or stability of D subgenome in the genetic background of the newly formed allopolyploids may depends on its genotypes (Mestiri et al., 2010), which accounts for why the D subgenome exhibited preferential elimination, while in another research, the D subgenome showed the highest stability as reported by Zhang et al. (2013a). Additionally, we found that the frequency of chromosome loss in the D^v^ genome from the *T. turgidum* × *Ae. ventricosa* cross was significantly higher than that observed in the *Ae. ventricosa* × *T. turgidum* cross. This may be due to the fact that the cytoplasm of the maternal parent gas different effects on different subgenomes. This finding suggests that although the D subgenome showed the greatest instability, allopolyploids containing D subgenome are likely to be maintained when the D subgenome donor was used as the maternal parent at its onset stage.

Small proportions of “hidden aneuploids” (0.78%) exhibiting the expected chromosomal number of parental euploids (2n = 56) with no discernible chromosomal structural alterations, but with loss and gain of chromosomes, were found. In the current study, none of the “hidden aneuploids” were found to involve homologous chromosomes, and the events of chromosomal loss/gain likely occurred randomly, which was consistent with the results described by Zhang et al. (2013a). The possible reason for this phenomenon is the allopolyploid nature of the wheat group.

We also observed large chromosomal structural changes, which is in line with the results reported by several studies (Hao et al., 2013; Fu et al., 2015; Su et al., 2015; Li G. et al., 2016; Guo et al., 2018). In contrast, few or no chromosomal structural changes have been identified in other studies (Mestiri et al., 2010; Zhao et al., 2011; Zhang et al., 2013a). In terms of the possible reasons for this discrepancy, it is possible that there was incompatibility between the subgenomes of the wheat group and the alien genomes (such as R genome), which would have led to the disorganization of meiotic pairing and given rise to chromosomal structural alterations. In addition, our findings revealed that the B subgenome showed the highest frequencies of chromosomal structural variation (6.04%), which was likely due to its high heterochromatin content and large genome size (Zhang et al., 2015).

In this study, we characterized the chromosomal behavior of early generations of the newly formed *T. turgidum*-*Ae. ventricosa* allopolyploids. Plants carrying numerical and structural chromosomal variations were found, indicating that genetic variations may occur on the post-allopolyploidization timescale. These variations are likely to confer rich adaptive abilities to individuals in various natural habitats, thus, fueling the establishment of new species (Han et al., 2015). As such, our results provide insight into the possible reasons for the existence of aneuploidy in the early generations of allopolyploidization in wheat group. Chromosome loss in the D^v^ subgenome showed cytoplasm-dependence, indicating a possible mechanism of allopolyploids harboring D subgenome. The synthetic amphiploids of the wheat group could also be used as a “bridge” for the transfer of valuable alien genes to wheat (Zhang et al., 2017; Zhang D. L. et al., 2018). In this respect, our *T. turgidum-Ae. ventricosa* amphiploids, possessing appealingly large seeds, could be used as a potential genetic resource for wheat improvement.

## Data Availability Statement

All datasets generated for this study are included in the article/Supplementary Material.

## Author Contributions

WY carried out the conceptualizaton. JZ and QW carried out the data curation. JZ, FY, YG, YW, and XZ performed the formal analysis. JZ, YG, and WY carried out the funding acquisition. JZ, YJ, and JL carried out the investigation. YW carried out the methodology. PX carried out the project administration. ZD performed the validation. JZ wrote the original draft. HW wrote, reviewed, and edited the manuscript.

## Conflict of Interest

The authors declare that the research was conducted in the absence of any commercial or financial relationships that could be construed as a potential conflict of interest.

## Supplementary Material

FIGURE S1 FISH analysis for metaphase spreads with chromosomal numerical variations. **(A–L)** Metaphase spreads with 46, 47, 48, 49, 50, 51, 52, 53, 54, 55, 56, and 57 chromosomes, respectively. 1s: Metaphase spread investigated using Oligo-pSc119.2 (green) and Oligo-pTa535 (red) as probes, 2s: Metaphase spreads using Oligo- (GAA)_7_ (yellow) as probe. Chromosomes were stained with DAPI (blue on 1s and red on 2s). Green denotes chromosome loss and yellow denotes showed chromosome gain. The two chromosomes lost are listed at the lower right corner of every images.

FIGURE S2 FISH analysis for metaphase spreads with chromosomal structural variations. **(A–T)** Metaphase spreads with 1AS. 1AL-, 3AL, -4AS.4AL, 7BS. 7BL-, 6AL, 1BL, 2BS. 2BL-, 4BL, 5BS, -6BS.6BL, 2D^v^L, 3D^v^L, 6D^v^S.6D^v^ L-, 1N^v^L, 3N^v^S.3N^v^ L-, 6N^v^S, 3BL.3BL, 5N^v^L.5N^v^L, 7A.7D^v^, 5AL.5AL, and 7N^v^L.3BL, respectively. 1s: Metaphase spread investigated using Oligo-pSc119.2 (green) and Oligo-pTa535 (red). 2s: Metaphase spreads investigated using Oligo- (GAA)_7_ (yellow). Chromosomes were stained with DAPI (blue on 1s and red on 2s). Red denotes type of chromosomal structural variations.
