# Supplementary figures and images for: Preferential Subgenome Elimination and Chromosomal Structural Changes Occurring in Newly Formed Tetraploid Wheat—Aegilops ventricosa Amphiploid (AABBDvDvNvNv)
Source: Front Genet. 2020 May 12;11:330. doi: 10.3389/fgene.2020.00330 (PMC7235383; doi:10.3389/fgene.2020.00330)

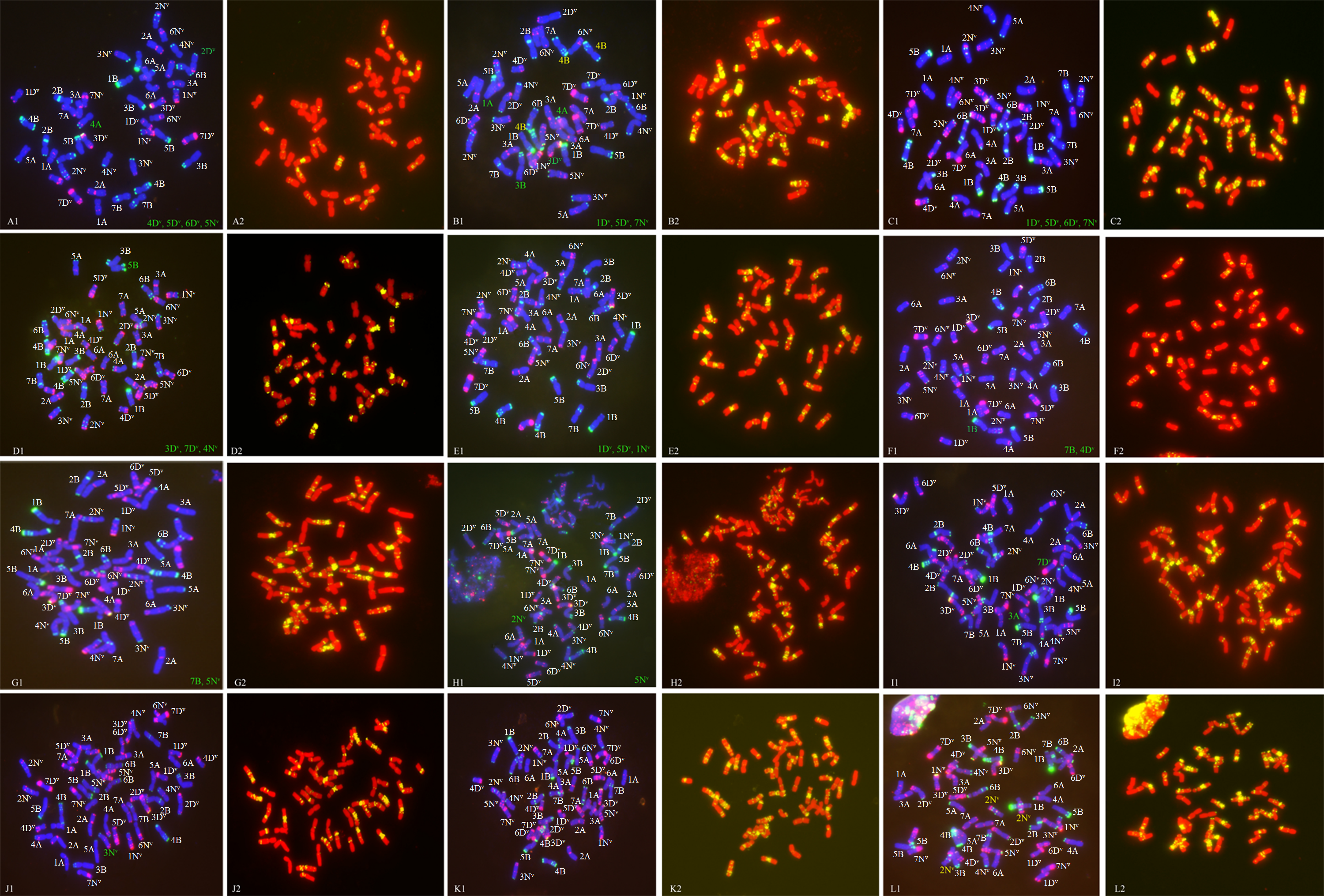

Supplement: FIGURE S1 — FISH analysis for metaphase spreads with chromosomal numerical variations. (A–L) Metaphase spreads with 46, 47, 48, 49, 50, 51, 52, 53, 54, 55, 56, and 57 chromosomes, respectively. 1s: Metaphase spread investigated using Oligo-pSc119.2 (green) and Oligo-pTa535 (red) as probes, 2s: Metaphase spreads using Oligo- (GAA)7 (yellow) as probe. Chromosomes were stained with DAPI (blue on 1s and red on 2s). Green denotes chromosome loss and yellow denotes showed chromosome gain. The two chromosomes lost are listed at the lower right corner of every images. [file Image_1.TIF]

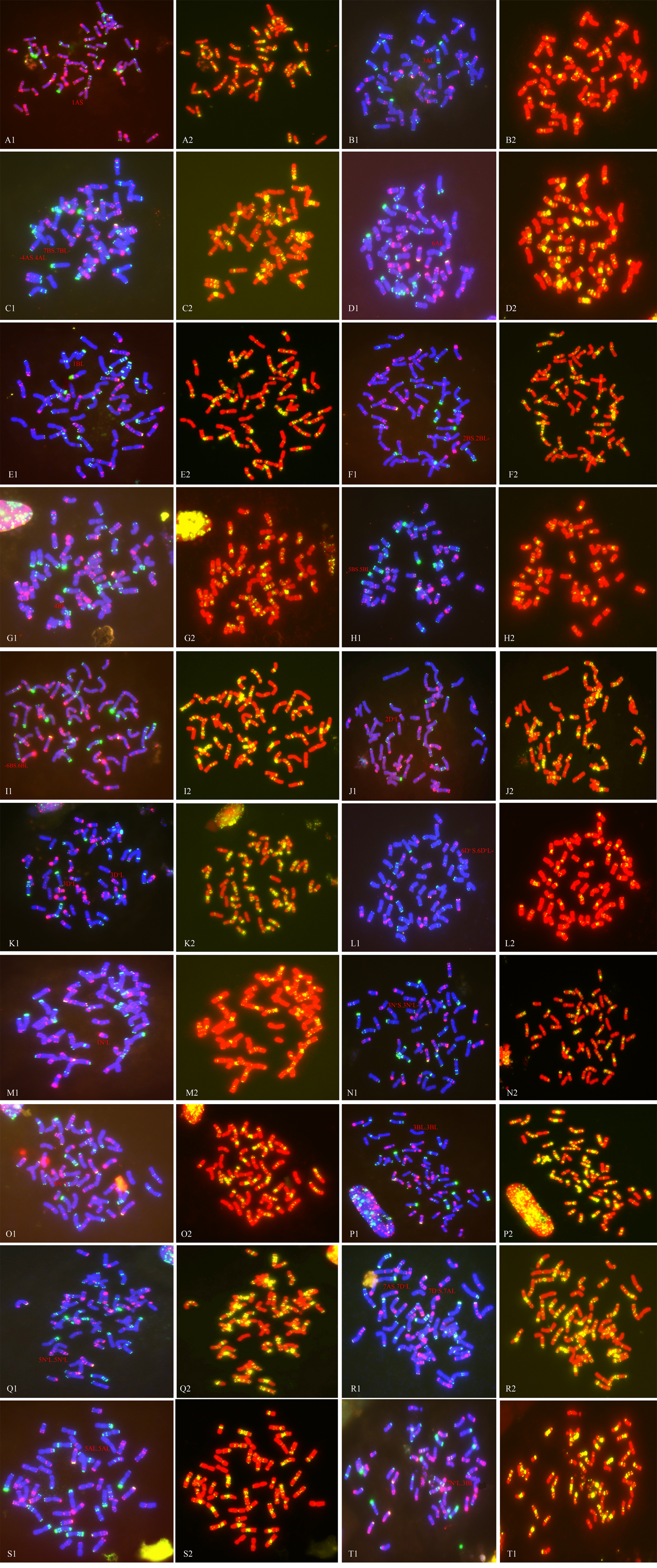

Supplement: FIGURE S2 — FISH analysis for metaphase spreads with chromosomal structural variations. (A–T) Metaphase spreads with 1AS. 1AL-, 3AL, -4AS.4AL, 7BS. 7BL-, 6AL, 1BL, 2BS. 2BL-, 4BL, 5BS, -6BS.6BL, 2DvL, 3DvL, 6DvS.6Dv L-, 1NvL, 3NvS.3Nv L-, 6NvS, 3BL.3BL, 5NvL.5NvL, 7A.7Dv, 5AL.5AL, and 7NvL.3BL, respectively. 1s: Metaphase spread investigated using Oligo-pSc119.2 (green) and Oligo-pTa535 (red). 2s: Metaphase spreads investigated using Oligo- (GAA)7 (yellow). Chromosomes were stained with DAPI (blue on 1s and red on 2s). Red denotes type of chromosomal structural variations. [file Image_2.TIF]
